# Supplementary material for: Pre-Germination Treatments at Operational Scale for Six Tree Species from the Sclerophyll Forest of Central Chile
Source: Plants (Basel). 2022 Feb 24;11(5):608. doi: 10.3390/plants11050608 (PMC8912697; doi:10.3390/plants11050608)
Supplement: Supplementary file 1 [file plants-11-00608-s001.zip › plants-1599021-supplementary.pdf]

Table S1. Selected species detailed description.

Species	Common name	Family	Origen/Distribution	Species characteristics	Conservation category	Uses	References
Quillaja saponaria Molina	Quillay	Quillajaceae	Native/Between 30° and 38°S	Broadleaf evergreen tree. 15 to 20 meters high. Leaves simple, alternate, elliptic or ovate, 3.5 cm long apex obtuse or subacute. Margin irregularly and shallowly toothed. Flowers white, 1.5 cm, 5-parted. Fruit 2.5 cm-wide, brown, a capsule that opens into a star shape. Flowering October to January; the dry fruits remain attached to the tree for a long time	Least concern	Bark contains saponin. Apiarian flower	[48] https://inaturalist.mma.gob.cl/taxa/181934-Quillaja-saponaria
Lithraea caustica Hook. & Arn.	Litre	Anacardiaceae	Endemic/Between 30° and 38°S	Broadleaf evergreen tree. 4 to 6 m height. Leaves simple, coriaceous, alternate. Margins oval to ovate with a rounded, acuminate or emarginate apex; upper surface green, glabrous, lower surface ferruginous, pilose or glabrous. Dioecious. Inflorescences paniculate, in the axils of upper leaves, rachis pubescent, 2–6 cm long. Flowers yellowish green, with short peduncles, unisexual. Fruit a drupe, 0.5–0.8 cm diameter, yellowish white and shiny. Flowering September to December, fruiting February to March	Least concern	Gastronomy and production of fermented beverages	[29,31] https://inaturalist.mma.gob.cl/taxa/543470-Lithraea-caustica
Acacia caven (Molina) Molina	Espino	*Fabaceae*	Native/Between 27° and 36°S	Evergreen shrub. 2 to 6 m height and 40 cm diameter. Branchlets with white stipular spines, 1–3 cm long. Leaves bipinnate, often in fascicles, bright green; pinnae 3–8	Least concern	Ornamental, perfumery and pharmaceutica. Production of carbon	[64] https://inaturalist.mma.gob.cl/t.

				pairs; pinnules 12–30 pairs. Inflorescence simple, axillary, with two to three heads per node; heads globular, 1–2 cm diameter, bright yellow, fragrant. Flowers densely packed, 5-merous, petals larger than sepals, stamens numerous. Legume cylindrical, 3–7 × 1.2–2.5 cm, leathery, dark brown or black. Flowering in spring, before the foliage emerges			axa/810560-Vachellia-caven
Porlieria chilensis I.M. Johnst.	Guayacán, palo santo	Zygophyllaceae	Endemic/Between 30° and 35°S	Small tree. Height up to 6 m. Leaves opposed, paripinnately compound, almost sessile, of 1.5 to 3.5 cm length, composed by 6 a 10 leaflets linear-oblong of complete margin and obtuse and mucronate apex. Single flowers, of 5 to 8 mm length, dark purple color. Sharp-pointed stipule. Fruit is a dehiscent capsule with 4 to 5 lobule, violet or dark purple in color.	Vulnerable	Craft, firewood, carbon and medicinal plant.	[36,52] https://clasificacionespecies.mma.gob.cl/wp-content/uploads/2019/10/Porlieria_chilensis_FINAL.pdf; https://clasificacionespecies.mma.gob.cl/wp-content/uploads/2019/10/Porlieria_chilensis_FINAL.pdf
Kageneckia angustifolia D. Don	Frangel, ovillo, pulpica, olivillo de la cordillera	Rosaceae	Endemic/Between 30 and 35°S	Semideciduous tree, dioecious. 4 to 7 m height, thin foliage. Lineal leaves or lineal-oblong, of 4-9 cm in length, coriaceous, bright, serrate, with teeth with glands and green-yellowish color ; 1-mm petioles. White flowers; masculine in axillary panicles. Fruit	Vulnerable	Ornamental	https://inaturalist.mma.gob.cl/taxa/716671-Kageneckia-angustifolia; https://clasificacionespecies.mm

				in capsule, woody, pubescent, and unequal locules. Small and numerous seeds, winged, imbricated and biseriate, laterally compressed.			a.gob.cl/wp-content/uploads/2019/10/Kageneckia_angustifolia_P08_corregida.pdf
Ceratonia chilensis Molina (Basonym: *Prosopis chilensis* (Molina) Stuntz emend. Burkart	Algarrobo	Fabaceae	Native/Between 18° and 35°S	Deciduous tree. 3-10 m height. Curved branches with spines. Hard spine, axillary, white, from caulinar origen of 6 to 10 cm length. Composed leaves, deciduous, glabrous, uni- bi- or tripinnated. Alternated fascicles between each pair of spines. Flowers in clusters disposed in groups of 2 to 4 in the same fascicles where leaves are formed. Fruit is a indehiscent legume, coriaceous with 12 to 18 cm length and 1 to 1.8 cm wide. Seeds have variable morphology with 20 to 32 seeds per fruit, brown-yellowish color, between 6 to 7 mm length.	Vulnerable	Ornamental, carpentry, forage production, medicinal, carbon and firewood.	[35] https://clasificacionespecies.mma.gob.cl/wp-content/uploads/2019/10/Prosopis_chilensis_2711.pdf; https://inaturalist.mma.gob.cl/taxa/327731-Prosopis-chilensis

Table S2. Pre-germination treatments applied by surveyed nurseries y reported by previous research for each of the evaluated species. N.R: Not reported

Species	Pre-germination treatments	Treatments applied by nurseries	Previous studies

Quillaja Saponaria	Without pretreatment (direct sowing)	Nursery 3.	N.R
	Soak in water	Nurseries 1, 2, 4, 5 and 6. (2 to 72 hours of soaking in water)	[51,68]
	Phytohormones (GA_3)	N.R	[42]
	Stratification	N.R	[46]
Lithraea caustica	Phytohormones (GA_3)	N.R	[69]
	Physical scarification	Nursery 2. (Hot water + 12 hours of soaking in water)	[69]
	Chemycal scarification	Nurseries 4, 5 and 6. (soaking for 30 to 90 minutes in sulfuric acid + soaking in water)	[29]
	Stratification	N.R	[69]
	Soak in Coke®	Nursery 1. (soaking in Coke for 4 days + soaking in water)	N.R
Acacia caven	Mechanical scarification	N.R	[62]
	Physical scarification	Nursery 2.	[46,67]
	Chemycal scarification	Nurseries 4, 5 and 6. (soaking for 90 to 180 minutes in sulfuric acid + soaking in water)	[37,45,62,63]
	Soak in Coke®	Nursery 1 (soaking in Coke for 4 days + soaking in water)	N.R
Porlieria chilensis	Soak in water	Nursery 4, 5 and 6. (2 to 72 hours of soaking in water)	[36]
	Phytohormones (GA_3)	N.R	[36]
	Physical scarification	N.R	[66]
	Stratification	N.R	[36,50,66]
Kageneckia angustifolia	Without pre-treatment (direct sowing)	N.R	[54]
	Soak in water	Nurseries 4, 5 and 6. (24 hours of soaking in water)	[51]

Ceratonia chilensis	Soak in water	Nursery 4.	N.R
	Physical scarification	Nursery 5.<br>(Hot water + 24 hours of soaking in water)	[51,65]
	Chemycal scarification	Nursery 6.<br>(Soaking for 30 minutes in sulfuric acid + soaking in water)	[65,68]
